# Identifying Activity Support Needs for individuals Aging With Disability: Subject Matter Expert Interviews

**DOI:** 10.1093/geroni/igaa057.2020

**Published:** 2020-12-16

**Authors:** Lyndsie Koon, Megan Bayles, Elena Remillard, Wendy Rogers

**Affiliations:** 1 University of Kansas, Lawrence, Kansas, United States; 2 University of Illinois, Champaign, Illinois, United States; 3 Georgia Institute Of Technology, Atlanta, Georgia, United States; 4 University of Illinois at Urbana-Champaign, Champaign, Illinois, United States

## Abstract

Technology designed to support aging-in-place for people with long-term disabilities begins with understanding the specific tasks that need support, and individual abilities, preferences, cultural practices, and privacy concerns. Such understanding is best achieved through a multi-method approach that includes direct, detailed assessments of representative users as well as individuals who work with or care for them. Our target users are people who identify as having a sensory or mobility impairment prior to the age of 50, including individuals aging with multiple sclerosis, late-onset hearing loss, and late-onset vision loss. In the present study, we are interviewing Subject Matter Experts (SMEs) to identify the scope of the challenges that should be explored in more depth. The SMEs include caregivers and medical professionals to identify challenges that the target populations experience in their everyday activities, advice about research adaptations, and recruitment ideas.

